# Publisher Correction: Truncated *FGFR2* is a clinically actionable oncogene in multiple cancers

**DOI:** 10.1038/s41586-022-05287-8

**Published:** 2022-09-01

**Authors:** Daniel Zingg, Jinhyuk Bhin, Julia Yemelyanenko, Sjors M. Kas, Frank Rolfs, Catrin Lutz, Jessica K. Lee, Sjoerd Klarenbeek, Ian M. Silverman, Stefano Annunziato, Chang S. Chan, Sander R. Piersma, Timo Eijkman, Madelon Badoux, Ewa Gogola, Bjørn Siteur, Justin Sprengers, Bim de Klein, Richard R. de Goeij-de Haas, Gregory M. Riedlinger, Hua Ke, Russell Madison, Anne Paulien Drenth, Eline van der Burg, Eva Schut, Linda Henneman, Martine H. van Miltenburg, Natalie Proost, Huiling Zhen, Ellen Wientjens, Roebi de Bruijn, Julian R. de Ruiter, Ute Boon, Renske de Korte-Grimmerink, Bastiaan van Gerwen, Luis Féliz, Ghassan K. Abou-Alfa, Jeffrey S. Ross, Marieke van de Ven, Sven Rottenberg, Edwin Cuppen, Anne Vaslin Chessex, Siraj M. Ali, Timothy C. Burn, Connie R. Jimenez, Shridar Ganesan, Lodewyk F. A. Wessels, Jos Jonkers

**Affiliations:** 1 Division of Molecular Pathology, Netherlands Cancer Institute, Amsterdam, The Netherlands; 2 Oncode Institute, Utrecht, The Netherlands; 3 Division of Molecular Carcinogenesis, Netherlands Cancer Institute, Amsterdam, The Netherlands; 4 OncoProteomics Laboratory, Department of Medical Oncology, Cancer Center Amsterdam, Amsterdam UMC, Vrije Universiteit Amsterdam, Amsterdam, The Netherlands; 5 Foundation Medicine, Cambridge, MA USA; 6 Experimental Animal Pathology, Netherlands Cancer Institute, Amsterdam, The Netherlands; 7 Incyte Research Institute, Wilmington, DE USA; 8 Department of Medicine, Division of Medical Oncology, Rutgers Cancer Institute of New Jersey, New Brunswick, NJ USA; 9 Department of Medicine and Pharmacology, Rutgers University, Piscataway, NJ USA; 10 Mouse Clinic for Cancer and Aging, Netherlands Cancer Institute, Amsterdam, The Netherlands; 11 Department of Pathology, Rutgers Cancer Institute of New Jersey, New Brunswick, NJ USA; 12 Incyte, Wilmington, DE USA; 13 Incyte Biosciences International, Morges, Switzerland; 14 Department of Medicine, Memorial Sloan Kettering Cancer Center, New York, NY USA; 15 Department of Medicine, Weill Medical College at Cornell University, New York, NY USA; 16 Upstate University Hospital, Upstate Medical University, Syracuse, NY USA; 17 Institute of Animal Pathology, Vetsuisse Faculty, University of Bern, Bern, Switzerland; 18 Bern Center for Precision Medicine, University of Bern, Bern, Switzerland; 19 Hartwig Medical Foundation, Amsterdam, The Netherlands; 20 Center for Molecular Medicine, University Medical Center Utrecht, Utrecht, The Netherlands; 21 Debiopharm International, Lausanne, Switzerland

**Keywords:** Breast cancer, Cancer models, Growth factor signalling, Oncogenes

Correction to: *Nature* 10.1038/s41586-022-05066-5 Published online 10 August 2022

This paper was originally published under a standard Springer Nature license (© The Author(s), under exclusive licence to Springer Nature Limited). It is now available as an open-access paper under a Creative Commons Attribution 4.0 International license, © The Author(s). The error has been corrected in the HTML and PDF versions of the article.

