# Supplementary material for: Truncated FGFR2 is a clinically actionable oncogene in multiple cancers
Source: Nature. 2022 Aug 10;608(7923):609–17. doi: 10.1038/s41586-022-05066-5 (PMC9436779; doi:10.1038/s41586-022-05066-5)
Supplement: Supplementary file 1 — Supplementary Figs. 1–2, Supplementary Tables 7–11 and the legends for Supplementary Tables 1–6. Supplementary Fig. 1: uncropped western blots related to Extended Data Figs. 5g and 7g. Supplementary Fig. 2: FACS gating strategies related to Extended Data Figs. 5a and 6b,c. Supplementary Table 7: the Fgfr2 GEMM cloning primer sequences. Supplementary Table 8: the mouse genotyping primer sequences. Supplementary Table 9: a list of the antibodies used. Supplementary Table 10: siRNA sequences. Supplementary Table 11: qPCR primer sequences. [file 41586_2022_5066_MOESM1_ESM.pdf]

nature portfolio

https://doi.org/10.1038/s41586-022-05066-5

Supplementary information

Truncated *FGFR2* is a clinically actionable oncogene in multiple cancers

In the format provided by the authors and unedited

Supplementary Information for Zingg, *et al.*

"Truncated *FGFR2* is a clinically actionable oncogene in multiple cancers"

Daniel Zingg[1,2,22], Jinhyuk Bhin[1,2,3,22], Julia Yemelyanenko[1,2,22], Sjors M. Kas[1,2,22], Frank Rolfs[1,2,4], Catrin Lutz[1,2], Jessica K. Lee[5], Sjoerd Klarenbeek[6], Ian M. Silverman[7], Stefano Annunziato[1,2], Chang S. Chan[8,9], Sander R. Piersma[4], Timo Eijkman[1,2], Madelon Badoux[1,2], Ewa Gogola[1,2], Bjørn Siteur[10], Justin Sprengers[10], Bim de Klein[1,2], Richard R. de Goeij-de Haas[4], Gregory M. Riedlinger[9,11], Hua Ke[8,9], Russell Madison[5], Anne Paulien Drenth[1,2], Eline van der Burg[1,2], Eva Schut[1,2], Linda Henneman[1,2,10], Martine H. van Miltenburg[1,2], Natalie Proost[10], Huiling Zhen[12], Ellen Wientjens[1,2], Roebi de Bruijn[1,2,3], Julian R. de Ruiter[1,2,3], Ute Boon[1,2], Renske de Korte-Grimmerink[10], Bastiaan van Gerwen[10], Luis Féliz[13], Ghassan K. Abou-Alfa[14,15], Jeffrey S. Ross[5,16], Marieke van de Ven[10], Sven Rottenberg[1,17,18], Edwin Cuppen[2,19,20], Anne Vaslin Chessex[21], Siraj M. Ali[5], Timothy C. Burn[7], Connie R. Jimenez[4], Shridar Ganesan[8,9,*], Lodewyk F. A. Wessels[2,3,*] & Jos Jonkers[1,2,*]

[1] Division of Molecular Pathology, Netherlands Cancer Institute, Amsterdam, The Netherlands.

[2] Oncode Institute, Utrecht, The Netherlands.

[3] Division of Molecular Carcinogenesis, Netherlands Cancer Institute, Amsterdam, The Netherlands.

[4] OncoProteomics Laboratory, Department Medical Oncology, Cancer Center Amsterdam, Amsterdam UMC, Vrije Universiteit Amsterdam, Amsterdam, The Netherlands.

[5] Foundation Medicine, Inc., Cambridge, MA, USA.

[6] Experimental Animal Pathology, Netherlands Cancer Institute, Amsterdam, The Netherlands.

[7] Incyte Research Institute, Wilmington, DE, USA.

[8] Department of Medicine, Division of Medical Oncology, Rutgers Cancer Institute of New Jersey, New Brunswick, NJ, USA.

[9] Rutgers University, Piscataway, NJ, USA.

[10] Mouse Clinic for Cancer and Aging, Netherlands Cancer Institute, Amsterdam, The Netherlands.

[11] Department of Pathology, Rutgers Cancer Institute of New Jersey, New Brunswick, NJ, USA.

[12] Incyte Corporation, Wilmington, DE, USA.

[13] Incyte Biosciences International Sàrl, Morges, Switzerland.

[14] Department of Medicine, Memorial Sloan Kettering Cancer Center, New York, NY, USA.

[15] Department of Medicine, Weill Medical College at Cornell University, New York, NY, USA.

[16] Upstate Medical University, Syracuse, NY, USA.

[17] Institute of Animal Pathology, Vetsuisse Faculty, University of Bern, Bern, Switzerland.

[18] Bern Center for Precision Medicine, University of Bern, Bern, Switzerland.

[19] Hartwig Medical Foundation, Amsterdam, The Netherlands.

[20] Center for Molecular Medicine, University Medical Center Utrecht, Utrecht, The Netherlands.

[21] Debiopharm International SA, Lausanne, Switzerland.

[22] These authors contributed equally: Daniel Zingg, Jinhyuk Bhin, Julia Yemelyanenko, Sjors M. Kas.

* e-mail: ganesash@cinj.rutgers.edu; l.wessels@nki.nl; j.jonkers@nki.nl

Supplementary Figure 1: Uncropped Western blots related to Extended Data Figs. 5g, 7g 3-4

Supplementary Figure 2: FACS gating strategies related to Extended Data Figs. 5a, 6b, c 5-6

Legend for Supplementary Table 1: List of *FGFR2* REs identified in the HMF cohort 7

Legend for Supplementary Table 2: List of *FGFR2* alterations identified in the FMI cohort 7

Legend for Supplementary Table 3: List of samples expressing *FGFR2*$^{\Delta E18}$ variants identified in TCGA cohort 7

Legend for Supplementary Table 4: Expression of lentiviral *FGFR2* constructs 7

Legend for Supplementary Table 5: List of *FGF/FGFR* alterations identified in CCLE cell lines with FGFRi responses 7

Legend for Supplementary Table 6: List of *FGF/FGFR* alterations identified in PDX models with Debio-1347 response 8

Supplementary Table 7: *Fgfr2* GEMM cloning primer sequences 8

Supplementary Table 8: Mouse genotyping primer sequences 8

Supplementary Table 9: Antibodies used 8

Supplementary Table 10: siRNA sequences 9

Supplementary Table 11: RT-qPCR primer sequences 9

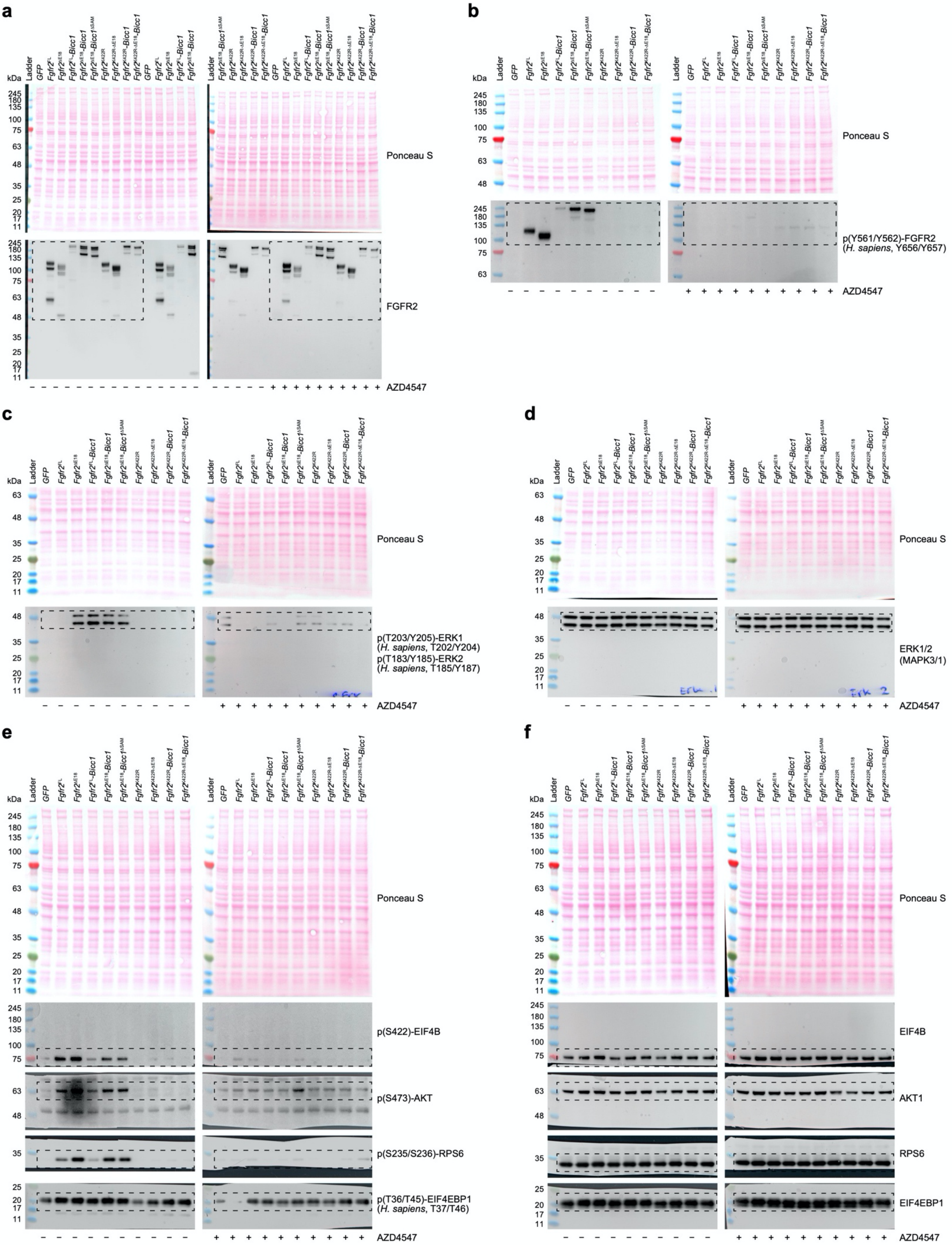
a
b
c
d
e
f
Ladder
GFP
kDa
Ponceau S
FGFR2
p(Y561/Y562)-FGFR2
(H. sapiens, Y656/Y657)
p(T203/Y205)-ERK1
(H. sapiens, T202/Y204)
p(T183/Y185)-ERK2
(H. sapiens, T185/Y187)
ERK1/2
(MAPK3/1)
p(S422)-EIF4B
p(S473)-AKT
p(S235/S236)-RPS6
p(T36/T45)-EIF4EBP1
(H. sapiens, T37/T46)
EIF4B
AKT1
RPS6
EIF4EBP1
AZD4547

g

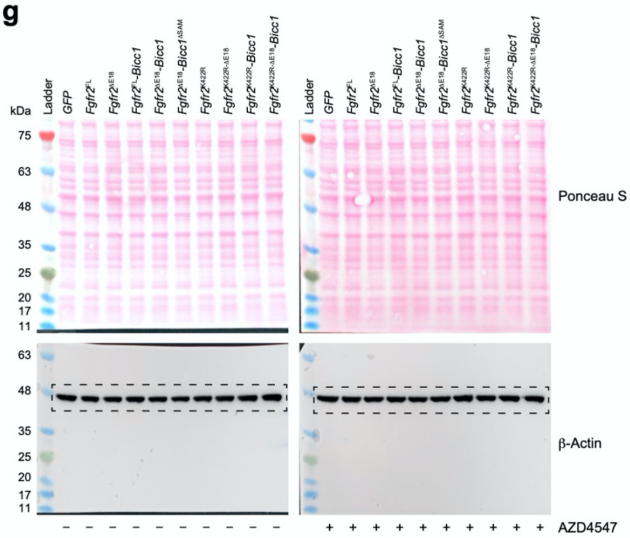

h

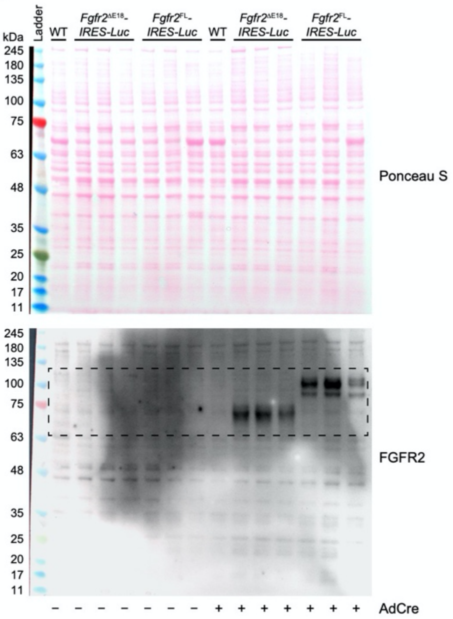

i

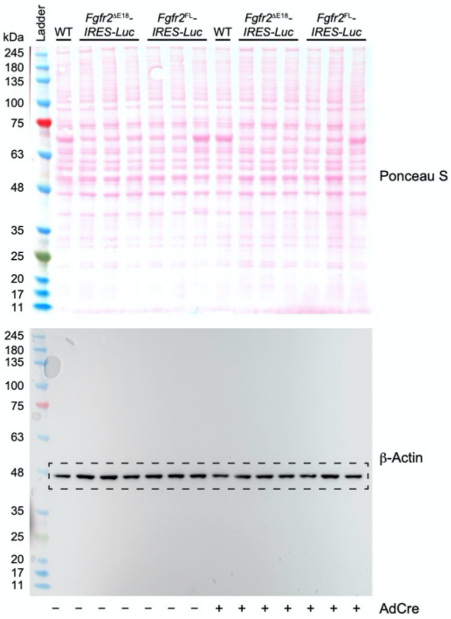

Supplementary Figure 1. Uncropped Western blots. a-**g**, Related to Extended Data Fig. 5g. Uncropped Western blots showing expression and phosphorylation of indicated proteins in NmuMG cells expressing *GFP* or indicated *Fgfr2* variants and treated for 3 hr with vehicle or 100 nM AZD4547. **h**, **i**, Related to Extended Data Fig. 7g. Uncropped Western blots showing FGFR2 and β-actin expression of mock-treated or AdCre-treated WT, *Fgfr2*^{FL}*-IRES-Luc*, or *Fgfr2*$^{\Delta E18}$*-IRES-Luc* MMEC cultures. Each blot was run with a BlueEye Prestained Protein Marker and stained with Ponceau S to ensure equal loading of total protein. Blots stained with the same antibody were developed and recorded in parallel and subjected to equal post-imaging processing. Black dashed squares indicate cropped bands.

a

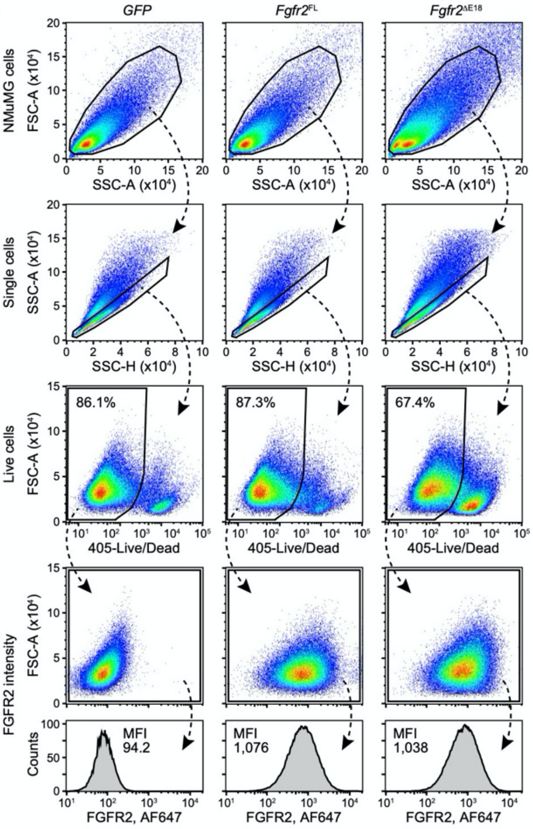
GFP
Fgfr2FL
Fgfr2ΔE18
NMuMG cells
FSC-A (x10⁴)
SSC-A (x10⁴)
Single cells
SSC-A (x10⁴)
SSC-H (x10⁴)
Live cells
FSC-A (x10⁴)
86.1%
87.3%
67.4%
405-Live/Dead
FGFR2 intensity
FSC-A (x10⁴)
Counts
MFI
94.2
MFI
1,076
MFI
1,038
FGFR2, AF647

b

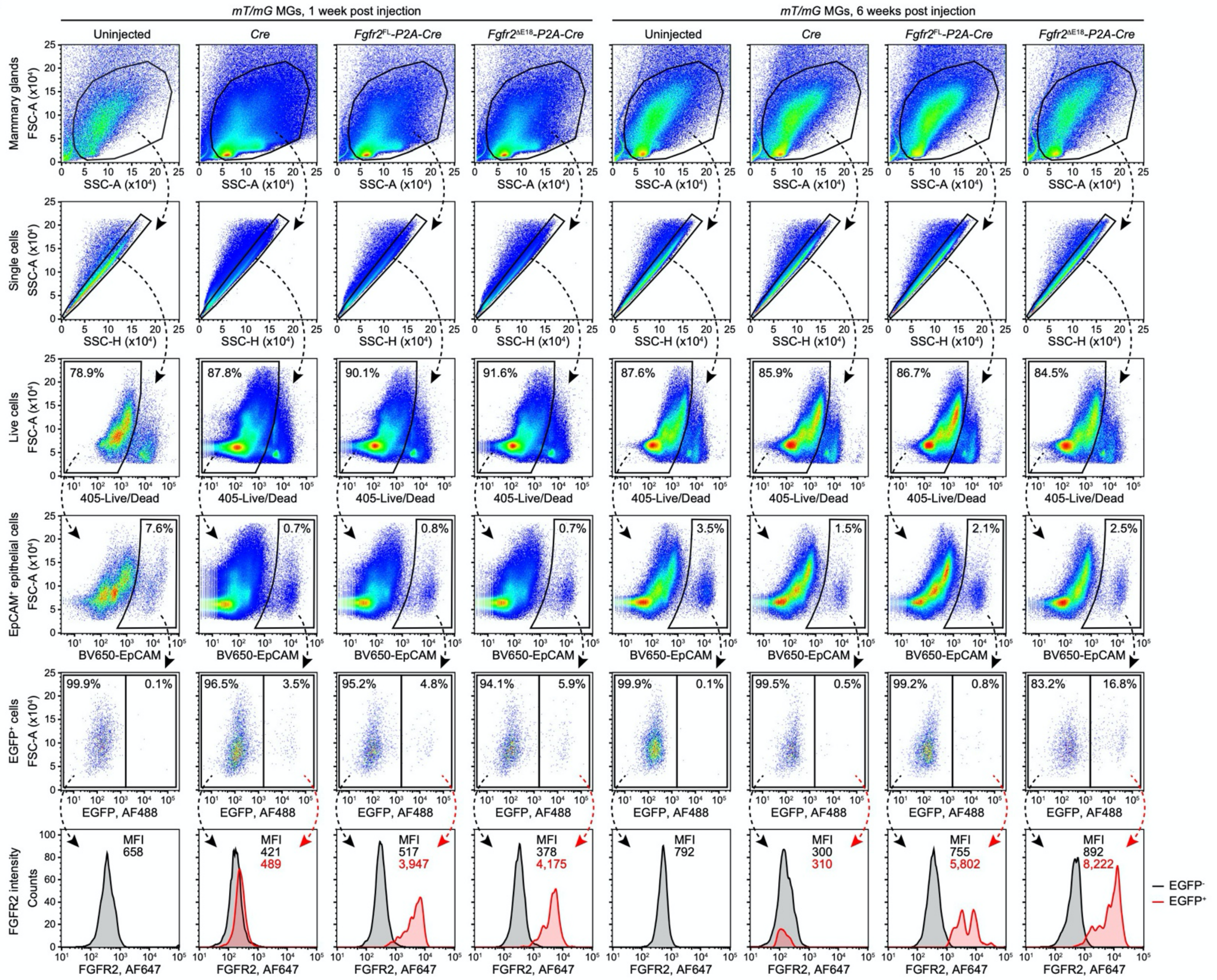

Supplementary Figure 2. FACS gating strategies. a, Related to Extended Data Fig. 5a. Fluorescence-activated cell sorting (FACS) gating strategy to analyse FGFR2 mean fluorescence intensity (MFI) in NmuMG cells expressing *GFP* or indicated *Fgfr2* variants. Cells were gated as follows: (i) FSC-A / SSC-A to select bulk of NmuMG cells and exclude debris events, (ii) SSC-A / SSC-H to select single cells, (iii) FSC-A / 405-Live/Dead to select live cells, and (iv) FSC-A / FGFR2, AF647 for gating to subsequently display FGFR2 intensity as histogram and measure FGFR2 MFI. **b**, Related to Extended Data Fig. 6b, c. FACS gating strategy to analyse traced EGFP$^+$ EpCAM$^+$ mammary epithelial cells and their FGFR2 MFI isolated form *Rosa26-mT/mG* female reporter mice intraductally injected with lentiviruses encoding *Cre* or indicated *Fgfr2-P2A-Cre* variants. Cre mediates *mT/mG* allele switching resulting in replacement of tdTomato (mT) expression by membrane-localised EGFP (mG) expression. Cells were gated as follows: (i) FSC-A / SSC-A to select bulk of mammary epithelial cell population and exclude debris events, (ii) SSC-A / SSC-H to select single cells, (iii) FSC-A / 405-Live/Dead to select live cells, (iv) FSC-A / BV650-EpCAM to select EpCAM$^+$ epithelial cells, (v) FSC-A / EGFP, AF488 to select and quantify EGFP$^-$ and EGFP$^+$ cells, and (vi) FSC-A / FGFR2, AF647 for gating (not shown) to subsequently display FGFR2 intensity as histogram and measure FGFR2 MFI of EGFP$^-$ and EGFP$^+$ cells.

Supplementary Table 1. List of *FGFR2* REs identified in the HMF cohort. List of *FGFR2* in-frame fusions and frame-unknown, intergenic, out-of-strand, and internal REs including BP information (genomic coordinates, corresponding genes, sequencing read orientations), CN information, and ploidy level information identified in 2,112 WGS profiles from HMF. Genomic coordinates of REs are denoted based on human reference genome GRCh37.

Supplementary Table 2. List of *FGFR2* alterations identified in the FMI cohort. Lists of *FGFR2* in-frame fusions and frame-unknown, intergenic, out-of-strand, and internal REs including dimerising capacity of RE partners as well as *FGFR2* E1-E17 partial amp and E1-E18 full-length amp and E18-splice-site, E18-truncating nonsense and frameshift, and missense hotspot mutations identified in diagnostic hybrid-capture panel-seq profiles from FMI (pan-cancer, n = 249,570; BRCA, n = 22,380). Alterations in top-30 co-altered driver genes are also disclosed. Genomic coordinates of REs are denoted based on human reference genome GRCh37. This table furthermore contains statistical details including exact P values, odds ratios, 95% confidence intervals, and FDR-adjusted P values corresponding to Fig. 3b, c and Extended Data Fig. 10b, c, e.

Supplementary Table 3. List of samples expressing *FGFR2*$^{\Delta E18}$ variants identified in TCGA cohort. List of *FGFR2* in-frame fusions and out-of-frame, intergenic and out-of-strand REs including BP information, amp status, and expression level (fusion fragments per million total reads, FFPM) of each RE as well as summary list of samples expressing *FGFR2*$^{\Delta E18}$ including REs, truncating mutations, and/or usage of E18-C3 or E18-C4, all identified in 10,344 samples from TCGA. Genomic coordinates of REs are denoted based on human reference genome GRCh38.

Supplementary Table 4. Expression of lentiviral *FGFR2* constructs. ***Sheet-1_NMuMG***, Expression of indicated lentiviral GFP-T2A-Puro and Fgfr2-T2A-Puro constructs in mouse NmuMG cells using RT-qPCR with primers spanning indicated cDNA segments. Expression of each cDNA segment is normalised to *Usf1* expression and to *Fgfr2* expression (average of *Fgfr2* E5–E6 and E17–E18) in *GFP*-expressing control cells. Data are represented as mean of n = 3 technical replica per group. Data represent 1 replica of 3 independent experiments. *Fgfr2*^{FL}, full-length (FL) *Fgfr2*. *Ate1*, *Bicc1*, and *Tacc2* correspond to the top-recurrent *ATE1*, *BICC1*, and *TACC2* fusion partner genes in Extended Data Fig. 2h. *Bicc1*$^{\Delta SAM}$ encodes BICC1 lacking its SAM oligomerisation domain. *Fgfr2*^{K422R} variants encode KD-dead FGFR2 variants. Truncated or alternative C-termini encoded by *IGR1/IGR2*, E18-C2/C3/C4, *Fgfr2*^{Y674*}, *Fgfr2*^{T678*}, *Fgfr2*^{P686*}, *Fgfr2*^{S694*}, *Fgfr2*^{V702*}, *Fgfr2*^{Y717*}, *Fgfr2*^{L681fs*6}, *Fgfr2*^{S687fs*3}, *Fgfr2*^{S697fs*4}, *Fgfr2*^{S704fs*22} are displayed in Extended Data Fig. 2k. *Fgfr2*^{S156W}, *Fgfr2*^{C287R}, *Fgfr2*^{N454K}, and *Fgfr2*^{K564E} correspond to the human *FGFR2*^{S252W}, *FGFR2*^{C382R}, *FGFR2*^{N549K}, and *FGFR2*^{K659E} missense hotspot mutations in Extended Data Fig. 2a. ***Sheet-2_KATO-III***, Expression of indicated lentiviral GFP-T2A-Puro and FGFR2-T2A-Puro constructs in KATO-III cells using RT-qPCR with primers spanning indicated cDNA segments. Expression of each cDNA segment is normalised to *USF1* expression and to *FGFR2* expression (average of *FGFR2* E4–E5 and E14–E16) in non-transduced WT MCF7 cells. Data are represented as mean of n = 3 technical replica per group of 1 experiment. *FGFR2*^{K517R} variants encode KD-dead FGFR2 variants. E4–E5 and E14–E16 primers detect endogenous *FGFR2* transcripts and *FGFR2* transcripts derived from lentiviral constructs. E1–E2 (5'-UTR), E18-C1 (3'-UTR), and E18-C3 (3'-UTR) primers specifically detect endogenous *FGFR2* transcripts. E18-C1–T2A, E17–T2A, and T2A–Puro primers specifically detect *FGFR2* transcripts derived from lentiviral constructs.

Supplementary Table 5. List of *FGF/FGFR* alterations identified in CCLE cell lines with FGFRi responses. List of *FGF/FGFR* amp status, *FGFR* missense hotspot mutations, *FGFR1-4* expression, *FGFR* composite expression, *FGFR2/3* REs, and/or usage of *FGFR2*–E18-C3 or E18-C4 in CCLE cell lines with FGFRi AUC values (AZD4547, n = 700; PD173074, n = 484).

Supplementary Table 6. List of *FGF/FGFR* alterations identified in PDX models with Debio-1347 response. List of *FGF/FGFR* amp status, *FGFR* missense hotspot mutations, *FGFR1-4* expression, *FGFR* composite expression, *FGFR2/3* REs, and/or usage of *FGFR2*–E18-C3 or E18-C4 in 36 CrownBio-HuPrime PDX models with Debio-1347 response data (disclosed as ΔT/ΔC values).

Supplementary Table 7. *Fgfr2* GEMM cloning primer sequences.

Target	Forward primer	Reverse primer
Fgfr2 variant amplification for *in vivo* shuttle vectors (FseI-PmeI)		
$Fgfr2^{FL}$	AAAAGGCCGGCCATGGGATTAC	AAAAGTTTAAACTCATGTTTTAACACTGCC
$Fgfr2^{\Delta E18}$	AAAAGGCCGGCCATGGGATTAC	AAAAGTTTAAACTCACTCATTGGTTGTGAG

Supplementary Table 8. Mouse genotyping primer sequences.

Target	Forward primer	Reverse primer
$Col1a1^{frt\text{-}invCAG\text{-}Fgfr2\text{-}FL\text{-}IRES\text{-}Luc}$ / $Col1a1^{frt\text{-}invCAG\text{-}Fgfr2\text{-}\Delta E18\text{-}IRES\text{-}Luc}$	TGGCCAGGGATATCAACAAC	ACACCGGCCTTATTCCAAGC
Col1a1 wild-type locus	CTCGCACGTACTTCATTC	CTGCTTGAATCCCTTTGAG

Supplementary Table 9. Antibodies used.

Target	Name	Provider	Identifier#	Lot#	Clone#	Dilution
FACS						
EpCAM	Rat monoclonal anti-EpCAM, BV650-conjugated	BD Biosciences	740559	1187955	G8.8	1:100
FGFR2	Rabbit monoclonal anti-FGFR2	Cell Signaling Technology	11835	4 and 5	D4H9	1:200
EGFP	Goat polyclonal anti-GFP	Abcam	ab6673	GR3371856-3	--	1:200
Goat-IgG	Donkey anti-goat IgG (H+L), AF488-conjugated	Thermo Fisher Scientific	A-11055	2301114	--	1:400
Rabbit-IgG	Donkey anti-rabbit IgG (H+L), AF647-conjugated	Thermo Fisher Scientific	A32795	WA308388	--	1:400
IHC						
MYC	Rabbit monoclonal anti-C-MYC	Abcam	ab32072	GR189790-46	Y69	1:4,000
Cyclin D1	Rabbit monoclonal anti-Cyclin D1	Abcam	ab16663	GR249365-2	SP4	1:200
E-cadherin	Rabbit monoclonal anti-E-cadherin	Cell Signaling Technology	3195	10	24E10	1:200
FGF3	Rabbit polyclonal anti-FGF3	LifeSpan BioSciences	LS-B11923	53099	--	1:100
FGFR2	Rabbit monoclonal anti-FGFR2	Cell Signaling Technology	11835	4 and 5	D4H9	1:200
PTEN	Rabbit monoclonal anti-PTEN	Cell Signaling Technology	9559	12	138G6	1:100
P53	Rabbit polyclonal anti-P53	Leica Biosystems	NCL-L-p53-CM5p	6070664	--	1:250
Phospho-Tyr IP						
p-Tyr-1000	Rabbit multi-monoclonal anti-p-Tyr mix	Cell Signaling Technology	8954	13	--	1:2,000
Western blotting						
β-Actin	Mouse IgG1 monoclonal anti-β-Actin	Sigma-Aldrich	A5441	127M4866V	AC-15	1:10,000
AKT1	Rabbit monoclonal anti-AKT1	Cell Signaling Technology	2938	4	C73H10	1:1,000
p(S473)-AKT	Rabbit monoclonal anti-p(S473)-AKT	Cell Signaling Technology	4060	25	D9E	1:1,000
EIF4B	Rabbit polyclonal anti-EIF4B	Cell Signaling Technology	3592	3	--	1:1,000
p(S422)-EIF4B	Rabbit polyclonal anti-p(S422)-EIF4B	Cell Signaling Technology	3591	6	--	1:1,000
EIF4EBP1	Rabbit monoclonal anti-EIF4EBP1	Cell Signaling Technology	9644	10	53H11	1:1,000
p(T37/T46)-EIF4EBP1	Rabbit monoclonal anti-p(T37/T46)-EIF4EBP1	Cell Signaling Technology	2855	17	236B4	1:1,000
ERK1/2	Rabbit monoclonal anti-ERK1/2	Cell Signaling Technology	4695	28	137F5	1:1,000
p(T202/Y204)-ERK1/2	Rabbit polyclonal anti-p(T202/Y204)-ERK1/2	Cell Signaling Technology	9101	30 and 31	--	1:1,000
FGFR2	Rabbit monoclonal anti-FGFR2	Cell Signaling Technology	11835	4 and 5	D4H9	1:1,000
p(Y653/Y654)-FGFR	Rabbit polyclonal anti-p(Y653/Y654)-FGFR	Cell Signaling Technology	3471	8 and 12	--	1:1,000
RPS6	Rabbit monoclonal anti-RPS6	Cell Signaling Technology	2217	10	5G10	1:1,000
p(S235/S236)-RPS6	Rabbit polyclonal anti-p(S235/S236)-RPS6	Cell Signaling Technology	2211	23	--	1:1,000
Mouse-IgG	Goat anti-mouse IgG (H+L), HRP-conjugated	Thermo Fisher Scientific	G-21040	1925065	--	1:20,000
Rabbit-IgG	Goat anti-rabbit IgG (H+L), HRP-conjugated	Dako	P0448	20083037	--	1:20,000

Supplementary Table 10. siRNA sequences.

Target	siRNA name	siRNA sequence
FGFR2 E5	siFGFR2^{E5}	GAAAAACGGGAAGGAGTTTtt
FGFR2 E9	siFGFR2^{E9}	CCTGTATGGTGGTAACAGTtt
FGFR2 E15	siFGFR2^{E15}	CCCTGTTTGATAGAGTATAtt
FGFR2 E18-C1	siFGFR2$^{E18\text{-}C1}$#1	GCTTGGACTTAACTAGTTAtt
FGFR2 E18-C1	siFGFR2$^{E18\text{-}C1}$#2	GGATGTCCCAATGCACCTAtt
FGFR2 E18-C3	siFGFR2$^{E18\text{-}C3}$#1	GCTTCATTGAGAAACTGGGtt
FGFR2 E18-C3	siFGFR2$^{E18\text{-}C3}$#2	GGCTTCATTGAGAAACTGGtt
FGFR2–COL14A1	siFGFR2-COL14A1#1	GAGCTACACATTGTTGTCAtt
FGFR2–COL14A1	siFGFR2-COL14A1#2	GTGAGACTTTGGTCAAAGTtt

Supplementary Table 11. RT-qPCR primer sequences.

Target	Forward primer	Reverse primer
Primers for mouse cDNA		
Fgfr2 E5–E6	TTAAGCAGGAGCATCGCATTG	GGCAGGTGTAGTTGCCTTTG
Fgfr2 E14–E15	GCCAGAAACGTGTTGGTAAC	TTCAGGAGCCATCCACTTG
Fgfr2 E17–E18 (used for *SB*-tumours)	GGATCGAATTCTGACTCTCAC	GGGTTCATAAGGCATGGG
Fgfr2 E17–E18 (used for cells)	CCACATTCAAGCAGTTGGTCG	AATACTGTTCGAGAGGCTGGG
Fgfr2 E17–*T2A*	CCACATTCAAGCAGTTGGTCG	TCGACGTCACCGCATGTTAG
Fgfr2 E18–*T2A* #1	TTTTCTCCAGACCCCATGCC	TCGACGTCACCGCATGTTAG
Fgfr2 E18–*T2A* #2	AATACTTGGATCTCACCCAGCC	TCGACGTCACCGCATGTTAG
Fgfr2 E17–*Ate1* E11	CCACATTCAAGCAGTTGGTCG	AATGGGCACCCAGACATACG
Fgfr2 E18–*Ate1* E11	TTTTCTCCAGACCCCATGCC	AATGGGCACCCAGACATACG
Ate1 E12–*T2A*	GCTGGAGTATGCAAACCTCGT	TCGACGTCACCGCATGTTAG
Fgfr2 E17–*Bicc1* E3	CCACATTCAAGCAGTTGGTCG	GCGTGTTTGTCTCCTCCATGA
Fgfr2 E18–*Bicc1* E3	TTTTCTCCAGACCCCATGCC	GCGTGTTTGTCTCCTCCATGA
Bicc1 E19–*T2A*	AGCGCAAGCAGAACAAATCAAG	TCGACGTCACCGCATGTTAG
Bicc1 E20–*T2A*	AAATGCTGCTGGCAATCTCAG	TCGACGTCACCGCATGTTAG
Fgfr2 E17–*Tacc2* E15	CCACATTCAAGCAGTTGGTCG	GAGACATCTGGTGGGTGAGC
Fgfr2 E18–*Tacc2* E15	TTTTCTCCAGACCCCATGCC	GAGACATCTGGTGGGTGAGC
Tacc2 E21–*T2A*	AAAGAACGCTGGAGCAGAAGA	TCGACGTCACCGCATGTTAG
Fgfr2 E17–*IGR1*	CCACATTCAAGCAGTTGGTCG	GAGCAGCATCCGCTGTTCT
IGR1–T2A	GCAATTCAGATGGGCAGAAGC	TCGACGTCACCGCATGTTAG
Fgfr2 E17–E18-C2	CCACATTCAAGCAGTTGGTCG	ATACGGTTCGAGAGGCTGAC
Fgfr2 E18-C2–*T2A*	CTCAGTCAGCCTCTCGAACC	TCGACGTCACCGCATGTTAG
Fgfr2 E17–E18-C4	CCACATTCAAGCAGTTGGTCG	CAGGTGAGAGGGGTTACATGG
Fgfr2 E18-C4–*T2A*	GCCACAAGCCACCATGTAAC	TCGACGTCACCGCATGTTAG
IRES–Luc	CCTCGGTGCACATGCTTTAC	CGCTGGGCCCTTCTTAATGT
Hprt	CTGGTGAAAAGGACCTCTCG	TGAAGTACTCATTATAGTCAAGGGCA
Usf1	CATCAGTGGTTACCCTGCCAC	ATCGTCACTGGTGAAAGCTCC
Primers for human cDNA		
FGFR2 E1–E2 (5'-UTR)	GAGGAGCGTTGCCATTCAAG	CACTTCCTCTACGGGCATGG
FGFR2 E4–E5 (CDS)	TGGTGCGGAAGATTTTGTCAG	AAACTTGACAGTGTTGGCCG
FGFR2 E14–E16 (CDS)	ACAAAAAGACCACCAATGGGC	TCCCACATTAACACCCCGAAG
FGFR2 E17–E18-C1 (3'-UTR)	CAACCAATGAGGAATACTTGGACC	ACTGAGGAAGGCATGGTTCG
FGFR2 E17–E18-C3 (3'-UTR)	CTCCCAGAGACCAACGTTCA	CCCAGTTTCTCAATGAAGCCATAA
FGFR2 E17–*COL14A1* E34	CCAGAGACCAACGTTCAAGC	TGACCAAAGTCTCACTGACAACA
COL14A1 E37–E41	CTGGCCAGCCTGGATATTGT	CAGCTCATCTTGGACAGGGG
FGFR2 E18-C1–*T2A*	TTTTCTCCAGACCCCATGCC	TCGACGTCACCGCATGTTAG
FGFR2 E17–*T2A*	TCCCAGAGACCAACGTTCAAG	TCGACGTCACCGCATGTTAG
USF1	ATGGAGAGCACCAAGTCTGG	TGGTTACTCTGCCGAAGCTC
Primers for mouse/human cDNA		
GFP–T2A	GTCCTGCTGGAGTTCGTGAC	TCGACGTCACCGCATGTTAG
T2A–Puro	AGGGCAGAGGAAGTCTGCTA	GGGGTAGTCGGCGAACG
