# Supplementary material for: Truncated FGFR2 is a clinically actionable oncogene in multiple cancers
Source: Nature. 2022 Aug 10;608(7923):609–17. doi: 10.1038/s41586-022-05066-5 (PMC9436779; doi:10.1038/s41586-022-05066-5)
Supplement: Supplementary file 2 — Reporting Summary [file 41586_2022_5066_MOESM2_ESM.pdf]

nature portfolio

Corresponding author(s): Shridar Ganesan, Lodewyk F. A. Wessels & Jos Jonkers

Last updated by author(s): Jun 17, 2022

Reporting Summary

Nature Portfolio wishes to improve the reproducibility of the work that we publish. This form provides structure for consistency and transparency in reporting. For further information on Nature Portfolio policies, see our Editorial Policies and the Editorial Policy Checklist.

Statistics

For all statistical analyses, confirm that the following items are present in the figure legend, table legend, main text, or Methods section.

n/a	Confirmed	
☐	☒	The exact sample size (*n*) for each experimental group/condition, given as a discrete number and unit of measurement
☐	☒	A statement on whether measurements were taken from distinct samples or whether the same sample was measured repeatedly
☐	☒	The statistical test(s) used AND whether they are one- or two-sided<br>*Only common tests should be described solely by name; describe more complex techniques in the Methods section.*
☒	☐	A description of all covariates tested
☐	☒	A description of any assumptions or corrections, such as tests of normality and adjustment for multiple comparisons
☐	☒	A full description of the statistical parameters including central tendency (e.g. means) or other basic estimates (e.g. regression coefficient) AND variation (e.g. standard deviation) or associated estimates of uncertainty (e.g. confidence intervals)
☐	☒	For null hypothesis testing, the test statistic (e.g. *F*, *t*, *r*) with confidence intervals, effect sizes, degrees of freedom and *P* value noted<br>*Give P values as exact values whenever suitable.*
☒	☐	For Bayesian analysis, information on the choice of priors and Markov chain Monte Carlo settings
☒	☐	For hierarchical and complex designs, identification of the appropriate level for tests and full reporting of outcomes
☐	☒	Estimates of effect sizes (e.g. Cohen's *d*, Pearson's *r*), indicating how they were calculated

Our web collection on statistics for biologists contains articles on many of the points above.

Software and code

Policy information about availability of computer code

Data collection

Sequencing data of Sleeping Beauty tumours was obtained from European Nucleotide Archive (ENA) with accession code PRJEB14134. WGS and RNA-seq data from HMF were downloaded from their google cloud computing platform under data sharing agreement DR-138. TCGAbiolinks (version 2.14.1; R package) was used to download TCGA raw RNA-seq data from GDC Data Portal (https://portal.gdc.cancer.gov/). CGP data were obtained from FMI via their standard data requesting process. For PDX models, genomic information and RNA-seq data was obtained from the CrownBio HuPrime data portal (https://www.crownbio.com/oncology/in-vivo-services/patient-derived-xenograft-pdx-tumor-models). Pharmacogenomic datasets of human cancer cell lines were obtained from CCLE (https://sites.broadinstitute.org/ccle/datasets), GDSC (https://www.cancerrxgene.org/), CTRP (https://portals.broadinstitute.org/ctrp.v2.1/), and PharmacoDB (https://www.pharmacodb.ca/) portals. FIGHT-202 oncogenomic data were obtained from Incyte via their standard data requesting process. Human reference genome for sequencing data analysis was obtained from the CTAT Genome Lib data resources (https://data.broadinstitute.org/Trinity/CTAT_RESOURCE_LIB/). Domain-domain interaction information of proteins was obtained from the 3did (https://3did.irbbarcelona.org) and the PPIDM (http://ppidm.loria.fr) databases. The SLIPPER Golden Standard Dataset of potentially (self)-interacting proteins was previously published by Liu, Z. et al. Proteome-wide prediction of self-interacting proteins based on multiple properties. Mol. Cell. Proteomics 12, 1689–700 (2013).

Data analysis

Analyses of in vitro and in vivo experiments were performed in Prism (version 9.3.1, GraphPad Software). All omics data analyses were performed in R (versions 3.6.3 - 4.1.2). Custom computer codes used to analyse the genomics and proteomics data in this study are available at https://doi.org/10.5281/zenodo.6630874 and https://doi.org/10.5281/zenodo.6630632, respectively.

Software used:
- Sample sizes calculation, G*Power software (version 3.1)
- IVIS measurements, Living Image Software (version 4.5.2, PerkinElmer)
- Digital processing of HE and IHC slides, CaseViewer software (version 2.2.1, 3DHISTECH)
- FACS analyses and quantifications thereof, BD FACSDiva Software (version 8.0.2, BD Biosciences) and FlowJo (version 10.7.1, BD Biosciences)
- Imaging of cell luminescence, fluorescence, or absorbance, Tecan i-control software (version 3.9.1, Tecan)

- 3D colony formation quantifiction, GelCount colony counting platform (version 1.1.2, Oxford Optronix)
- Primer design for In-Fusion, SnapGene (version 5.2)
- Primer design for site-directed mutagenesis, QuikChange Primer Design (https://www.agilent.com/store/primerDesignProgram.jsp)
- Primer design for RT-qPCR, Primer-BLAST (https://www.ncbi.nlm.nih.gov/tools/primer-blast/)
- RT-qPCR recording, QuantStudio Real-Time PCR Software (version 1.7.2, Thermo Fisher Scientific)
- Western blot recording , Fusion FX7 Edge imaging system (version 18.05, Vilber)
- Western blot post-imaging processing, Photoshop 2022 (version 23.2.2, Adobe)
- Western blot band intensity quantification, Fiji (version 1.0)
- LC-MS/MS operation, Tune (version 2.11) and Xcalibur Software (version 4.3.73.11, #OPTON-30965, both Thermo Fisher Scientific)
- HiSeq 2500 System operation, HiSeq Control Software (version 2.2.68, Illumina)
- NextSeq 500 System operation, NextSeq Control Software (version 2.0.2, Illumina)
- NovaSeq 6000 System operation, NovaSeq Control Software (version 1.7.5, Illumina)

Software/packages used for proteomics analyses:
- MS/MS spectra annotation, MaxQuant software (version 2.0.3.0) with default settings and the Swissprot M. musculus reference proteome
- Spectral library generation from MaxQuant output file, Spectronaut software (version 15.4.210913, Biognosys)
- Single sample gene set enrichment analysis (ssGSEA), via GenePattern platform (https://www.genepattern.org) using the ssGSEA module (version 10.0.11) and hallmark gene sets from MSigDB (version 7.0)
- Differential expression analysis on class 1 phosphosite intensity data, R package limma (version 3.52.1)
- (Single sample) phosphosite signature enrichment analysis ((ss)PTM-SEA), via GenePattern platform (https://www.genepattern.org) using a 7-AA sequence flanking the phosphosite as identifier and the murine kinase/pathway definitions of PTMsigDB (version 1.9.0)
- Robust kinase activity inference (RoKAI) tool (https://rokai.io), used with default settings and the Uniprot M. musculus reference proteome

Software/packages used for genomics analyses:
- Analysis of WGS data from HMF, published computer code at https://github.com/hartwigmedical/pipeline5
- WGS read mapping, BWA-MEM (version 0.7.5a) and the human reference genome GRCh37
- WGS somatic SV calling, GRIDSS (version 1.8.0)
- WGS CNA and tumour purity estimation, PURPLE (version 2.43)
- WGS event annotation and derivate chromosome construction, LINX (version 1.9)
- Low-coverage WGS read mapping, BBWA-MEM (version 0.7.5a) and the human reference genome GRCh38
- Low-coverage WGS alignment analysis, QDNAseq (version 1.14.0) with 20,000 bp bin size
- TCGA raw RNA-seq data download from GDC Data Portal (https://portal.gdc.cancer.gov/), TCGAbiolinks (version 2.14.1; R package)
- RNA-seq read mapping, STAR (version 2.7.2) and STAR-Fusion (version 1.8.1) and the human reference genome GRCh38 gencode v32 CTAT
- RNA-seq gene and exon level expression quantification, featureCounts (version 1.6.2)
- RNA-seq read count normalisation, Trimmed Mean of M-value (TMM) method via edgeR (version 3.26.6)
- RNA-seq sashimi plots, Integrated Genomics Viewer (version 1.11.0, https://igv.org/app/).
- hybrid-capture RNA-seq read mapping, STAR (version 2.7.3a), RSEM (version 1.3.0) and LeafCutter (version 0.2.9)
- PDX-derived RNA-seq mapping, Disambiguate (version 2018.05.03-6) to filter mouse (mm10 gencode M23) from human (GRCh38 gencode v32 CTAT)-derived reads
- Genome coordinates conversion from GRCh37 to GRCh38, UCSC Lift Genome Annotations (https://genome.ucsc.edu/cgi-bin/hgLiftOver)
- Self-interacting capacity of FGFR2 RE partners, SLIPPER algorithm (Liu et al., 2013, Proteome-wide prediction of self-interacting proteins based on multiple properties)
- Domain enrichment analysis among FGFR2 RE partners, DAVID bioinformatic resources (https://david.ncifcrf.gov/)

For manuscripts utilizing custom algorithms or software that are central to the research but not yet described in published literature, software must be made available to editors and reviewers. We strongly encourage code deposition in a community repository (e.g. GitHub). See the Nature Portfolio guidelines for submitting code & software for further information.

Data

Policy information about availability of data

All manuscripts must include a data availability statement. This statement should provide the following information, where applicable:

- Accession codes, unique identifiers, or web links for publicly available datasets
- A description of any restrictions on data availability
- For clinical datasets or third party data, please ensure that the statement adheres to our policy

All data generated or analysed during this study are included in this article and its Supplementary Information. Source data are provided with this paper.

- The low-coverage WGS and RNA-seq data of human cell lines generated in this study are available in the European Nucleotide Archive (ENA) under accession number PRJEB42514
- The mass spectrometry proteomics data and MaxQuant-generated text files generated in this study are available in the ProteomeXchange Consortium under accession numbers PXD031711 (Fgfr2 samples) and PXD032007 (KB1P(M) samples)
- Sequencing data of SB-tumours were previously published (Kas et al., 2017) and are available in ENA under accession number PRJEB14134
- WGS and RNA-seq data from HMF can be obtained through standardized procedures and request forms at https://www.hartwigmedicalfoundation.nl/en/
- CGP data can be obtained from FMI on reasonable request at https://www.foundationmedicine.com/service/genomic-data-solutions
- Data from TCGA, CCLE, CTRPv2, and GDSC are available through the respective data portals at https://portal.gdc.cancer.gov/, https://sites.broadinstitute.org/ccle/datasets, https://www.cancerrxgene.org/, https://portals.broadinstitute.org/ctrp.v2.1/, and https://www.pharmacodb.ca/
- Details on PDXs can be obtained from the CrownBio-HuPrime data portal at https://www.crownbio.com/oncology/in-vivo-services/patient-derived-xenograft-pdx-tumor-models.
- The FIGHT-202 study was previously published (Abou-Alfa et al., 2020). Information on Incyte's clinical trial data sharing policy and instructions for submitting clinical trial data requests are available at https://www.incyte.com/Portals/0/Assets/Compliance%20and%20Transparency/clinical-trial-data-sharing.pdf?ver=2020-05-21-132838-960
- Human reference genome (GRCh38 gencode v32 CTAT) used for RNA-seq data analysis is available in CTAT Genome Lib data resources at https://data.broadinstitute.org/Trinity/CTAT_RESOURCE_LIB
- SLIPPER list of self-interacting proteins was previously published (Liu et al., 2013, Proteome-wide prediction of self-interacting proteins based on multiple

properties)
- 3did and PPIDM domain-domain interaction information is available at https://3did.irbbarcelona.org and http://ppidm.loria.fr
- Reference human and mouse Swissprot proteome information is available in the UniProt database at https://www.uniprot.org

Field-specific reporting

Please select the one below that is the best fit for your research. If you are not sure, read the appropriate sections before making your selection.

☒ Life sciences ☐ Behavioural & social sciences ☐ Ecological, evolutionary & environmental sciences

For a reference copy of the document with all sections, see nature.com/documents/nr-reporting-summary-flat.pdf

Life sciences study design

All studies must disclose on these points even when the disclosure is negative.

Sample size	Sample size determinations for the FIGHT-202 (NCT02924376) clinical trial were previously described (Abou-Alfa et al., 2020), and were large enough to measure the effect sizes. Sample sizes in the SB-transposon insertional mutagenesis screen and ex vivo analyses thereof were previously published (Kas et al., 2017), and were large enough to measure the effect sizes. Sample sizes for the remaining in vivo experiments and follow-up ex vivo experiments were determined using G*Power software (version 3.1) and were large enough to measure the effect sizes. For in vitro experiments, no sample size calculations were performed. Instead, in vitro experiments' sample sizes were based on previous experiences. For all in vitro experiments, large enough sample sizes were obtained to appropriately evaluate statistical differences between experimental groups and to ensure reproducibility.
Data exclusions	No data had to be excluded.
Replication	For in vitro global expression proteomics and global phosphoproteomics using IMAC enrichment, 4 independent NMuMG cell replica per Fgfr2 variant tested were collected and subjected to the two methodologies in parallel. An independent set of 3 replica per variant was collected for -Tyr-specific proteomics using p-Tyr-IP enrichment. Sample processing and LC-MS/MS measurements were performed across 3 randomized batches per methodology. One global phosphoproteomic sample had to be excluded because of low protein concentration (Fgfr2-dE18-Bicc1_4).<br>Other in vitro experiments were repeated independently at least twice, and all attempts at replication were successful. Across these independent experiments, data on a total of at least 3 independent replica were collected.<br>In vivo tumorigenesis and intervention studies were each performed as 'single' experiments, but in sufficient mice (= independent biological replica) to measure the effect sizes. For intraductual injections of lentiviruses, virus variants were injected in parallel in several batches across several days, rather than injection each virus in series. No obvious differences in tumour latencies were observed in between 'replica' of injection. For the intervention studies, tumour-bearing mice were continuously randomly allocated to treatment arms when tumours reached treatment-starting size. No obvious response differences were observed in between mice with faster versus slower growing tumours. All attempts at replication were successful.<br>Ex vivo H&E + IHC stainings and analyses of mouse mammary tissues was performed in multiple independent batches. For each tissue sample, one H&E and/or IHC stain per marker was evaluated. All attempts at replication were successful.<br>Ex vivo FACS analyses were done on pooled mammary gland samples from at least 4 different mice per time-point analysed. Typically, per batch of analysis 1-2 mice per Fgfr2 variant from one time-point were analysed. All attempts at replication were successful.<br>Ex vivo proteomics was performed on individual mouse mammary tumours subjecting each sample to three (phospho)-proteomic methodologies in parallel (global expression proteomics, global phosphoproteomics using IMAC enrichment, and p-Tyr-specific proteomics using p-Tyr-IP enrichment). Sample processing and LC-MS/MS measurements were performed across several randomized batches. A few p-Tyr-IP samples hat to be excluded because of low protein concentrations (Fgfr2-FL_1, Fgfr2-FL-Ate1_1, Fgfr2-FL-Bicc1_2, Fgfr2-FL-Tacc2_2, Fgfr2-dE18-Ate1_2, Fgfr2-dE18-Tacc2_2, Fgfr2-E18-C4_1).
Randomization	Allocation of mice into lentivirus injection cohorts, tumour fragment transplantation cohorts, as well as into treatment arms was randomized. For DNA/RNA-seq and proteomics experiments, samples/replica were randomized during processing and data acquisition. For other experiments, no randomization strategy was applied.
Blinding	Animal care takers and animal pathologists were actively blinded towards mouse examinations and histopathological evaluations. For other experiments, no active blinding strategy was applied. Yet, experiments were performed by a multitude of researchers and technicians, the majority of whom were agnostic to the outcome of experiments.

Reporting for specific materials, systems and methods

We require information from authors about some types of materials, experimental systems and methods used in many studies. Here, indicate whether each material, system or method listed is relevant to your study. If you are not sure if a list item applies to your research, read the appropriate section before selecting a response.

Materials & experimental systems

n/a	Involved in the study
☐	☒ Antibodies
☐	☒ Eukaryotic cell lines
☒	☐ Palaeontology and archaeology
☐	☒ Animals and other organisms
☐	☒ Human research participants
☐	☒ Clinical data
☒	☐ Dual use research of concern

Methods

n/a	Involved in the study
☒	☐ ChIP-seq
☐	☒ Flow cytometry
☒	☐ MRI-based neuroimaging

Antibodies

Antibodies used

Mouse IgG1 monoclonal anti-β-Actin, Sigma-Aldrich, #A5441, lot#127M4866V, clone#AC-15
Rabbit monoclonal anti-AKT1, Cell Signaling Technology, #2938, lot#4, clone#C73H10
Rabbit monoclonal anti-p(S473)-AKT, Cell Signaling Technology, #4060, lot#25, clone#D9E
Rabbit monoclonal anti-E-cadherin, Cell Signaling Technology, #3195, lot#10, clone#24E10
Rabbit monoclonal anti-Cyclin D1, Abcam, #ab16663, lot#GR249365-2, clone#SP4
Rabbit polyclonal anti-EIF4B, Cell Signaling Technology, #3592, lot#3
Rabbit polyclonal anti-p(S422)-EIF4B, Cell Signaling Technology, #3591, lot#6
Rabbit monoclonal anti-EIF4EBP1, Cell Signaling Technology, #9644, lot#10, clone#53H11
Rabbit monoclonal anti-p(T37/T46)-EIF4EBP1, Cell Signaling Technology, #2855, lot#17, clone#236B4
Rat monoclonal anti-EpCAM, BV650-conjugated, BD Biosciences, #740559, lot#1187955, clone#G8.8
Rabbit monoclonal anti-ERK1/2, Cell Signaling Technology, #4695, lot#28, clone#137F5
Rabbit polyclonal anti-p(T202/Y204)-ERK1/2, Cell Signaling Technology, #9101, lot#30 and 31
Rabbit polyclonal anti-FGF3, LifeSpan BioSciences, #LS-B11923, lot#53099
Rabbit monoclonal anti-FGFR2, Cell Signaling Technology, #11835, lot#4 and 5, clone#D4H9
Rabbit polyclonal anti-p(Y653/Y654)-FGFR, Cell Signaling Technology, #3471, lot#8 and 12
Goat polyclonal anti-GFP, Abcam, #ab6673, lot#GR3371856-3
Rabbit monoclonal anti-C-MYC, Abcam, #ab32072, lot#GR189790-46, clone#Y69
Rabbit polyclonal anti-P53, Leica Biosystems, #NCL-L-p53-CM5p, lot#6070664
Rabbit monoclonal anti-PTEN, Cell Signaling Technology, #9559, lot#12, clone#138G6
Rabbit monoclonal anti-RPS6, Cell Signaling Technology, #2217, lot#10, clone#5G10
Rabbit polyclonal anti-p(S235/S236)-RPS6, Cell Signaling Technology, #2211, lot#23
Rabbit multi-monoclonal anti-p-Tyr mix, Cell Signaling Technology, #8954, lot#13
Donkey polyclonal anti-goat IgG (H+L), AF488-conjugated, Thermo Fisher Scientific, #A-11055, lot#2301114
Donkey polyclonal anti-rabbit IgG (H+L), AF647-conjugated, Thermo Fisher Scientific, #A32795, lot#WA308388
Goat polyclonal anti-mouse IgG (H+L), HRP-conjugated, Thermo Fisher Scientific, #G-21040, lot#1925065
Goat polyclonal anti-rabbit IgG (H+L), HRP-conjugated, Dako, #P0448, lot#20083037

Validation

Primary antibodies used for IHC were rabbit monoclonal anti-E-cadherin (CST #3195), rabbit monoclonal anti-Cyclin D1 (Abcam #ab16663), rabbit polyclonal anti-FGF3 (LifeSpan #LS-B11923), rabbit monoclonal anti-FGFR2 (CST #11835), rabbit monoclonal anti-C-MYC (Abcam #ab32072), rabbit polyclonal anti-P53 (Leica Biosystems #NCL-L-p53-CM5p), and rabbit monoclonal anti-PTEN (CST #9559). The antibodies were independently validated by a certified pathologist by evaluation of IHC results in positive and negative biological control FFPE tissues to ensure specificity and sensitivity. In addition, negative technical controls were performed by omission of the primary antibody in extra sections for a randomly selected small subset of the samples.

The primary antibody BV650-conjugated rat monoclonal anti-EpCAM (BD Biosciences #740559) used for FACS was validated by BD Biosciences (https://www.bdbiosciences.com/en-ca/products/reagents/flow-cytometry-reagents/research-reagents/single-color-antibodies-ruo/purified-rat-anti-mouse-cd326.552370) for this application. The primary antibodies goat anti polyclonal anti-GFP (Abcam #ab6673) and rabbit monoclonal anti-FGFR2 (CST #11835) were validated for FACS using cells overexpressing EGFP or FGFR2 versus control cells negative for GFP or FGFR2. These controls were taken along for each FACS experiment on mouse derived mammary glands and in vitro-cultured NMuMG cells.

The primary control antibody used for Western blotting was mouse IgG1 monoclonal anti-β-Actin (Sigma-Aldrich #A5441) and has been validated by Sigma-Aldrich for this application (https://www.sigmaaldrich.com/NL/en/product/sigma/a5441).
All other primary antibodies used for Western blotting or p-Tyr IPs were derived from Cell Signaling Technology (CST) and were validated for specificity and sensitivity in the respective applications by CST according to their rigid Antibody Validation Principles (https://www.cellsignal.com/about-us/cst-antibody-validation-principles). Details on each antibody and its validation data are in the following links:
Rabbit monoclonal anti-AKT1 (CST #2938, https://www.cellsignal.com/products/primary-antibodies/akt1-c73h10-rabbit-mab/2938)
Rabbit monoclonal anti-p(S473)-AKT (CST #4060, https://www.cellsignal.com/products/primary-antibodies/phospho-akt-ser473-d9e-xp-rabbit-mab/4060)
Rabbit polyclonal anti-EIF4B (CST #3592, https://www.cellsignal.com/products/primary-antibodies/eif4b-antibody/3592)
Rabbit monoclonal anti-EIF4EBP1 (CST #9644, https://www.cellsignal.com/products/primary-antibodies/4e-bp1-53h11-rabbit-mab/9644)
Rabbit monoclonal anti-p(T37/T46)-EIF4EBP1 (CST #2855, https://www.cellsignal.com/products/primary-antibodies/phospho-4e-bp1-thr37-46-236b4-rabbit-mab/2855)
Rabbit monoclonal anti-ERK1/2 (CST #4695, https://www.cellsignal.com/products/primary-antibodies/p44-42-mapk-erk1-2-137f5-rabbit-mab/4695)
Rabbit polyclonal anti-p(T202/Y204)-ERK1/2 (CST #9101, https://www.cellsignal.com/products/primary-antibodies/phospho-p44-42-

mapk-erk1-2-thr202-tyr204-antibody/9101)
Rabbit monoclonal anti-FGFR2 (CST #11835, https://www.cellsignal.com/products/primary-antibodies/fgf-receptor-2-d4h9-rabbit-mab/11835)
Rabbit polyclonal anti-p(Y653/Y654)-FGFR (CST #3471. https://www.cellsignal.com/products/primary-antibodies/phospho-fgf-receptor-tyr653-654-antibody/3471)
Rabbit monoclonal anti-RPS6 (CST #2217, https://www.cellsignal.com/products/primary-antibodies/s6-ribosomal-protein-5g10-rabbit-mab/2217)
Rabbit polyclonal anti-p(S235/S236)-RPS6 (CST #2211, https://www.cellsignal.com/products/primary-antibodies/phospho-s6-ribosomal-protein-ser235-236-antibody/2211)
Rabbit multi-monoclonal anti-p-Tyr mix (CST #8954, https://www.cellsignal.com/products/primary-antibodies/phospho-tyrosine-p-tyr-1000-multimab-rabbit-mab-mix/8954)

Eukaryotic cell lines

Policy information about cell lines

Cell line source(s)	HEK 293T cells (#CRL-3216, ATCC), MCF7 (#HTB-22, ATCC), MDA-MB-134-VI (#HTB-23, ATCC), MDA-MB-231 (#HTB-26, ATCC), NCI-H716 (#CCL-251, ATCC), NMuMG (#CRL-1636, ATCC), KATO-III (#HTB-103, ATCC), SNU-1 (#CRL-5971, ATCC), SNU-16 (#CRL-5974, ATCC), MFM-223 (#98050130, ECACC), and SUM52PE (#HUMANSUM-0003018, BioIVT).
Authentication	Cell lines were previously authenticated by providers. No re-authentication was performed for this study.
Mycoplasma contamination	Routine mycoplasma testing repeatedly confirmed all cell lines used to be negative for mycoplasma via the MycoAlert Mycoplasma Detection Kit (#LT07-218, Lonza).
Commonly misidentified lines (See ICLAC register)	No commonly misidentified cell lines were used in this study.

Animals and other organisms

Policy information about studies involving animals; ARRIVE guidelines recommended for reporting animal research

Laboratory animals	Mice, all female.<br>- GEMMs and somatic mouse models: FVB/NRj background, 6-week-old. Strains: WT, Wap-Cre;Cdh1F/F, Wap-Cre;Cdh1F/F;Fgfr2-FL-IRES-Luc, Wap-Cre;Cdh1F/F;Fgfr2-dE18-IRES-Luc, Trp53F/F, Trp53F/F;Rosa26-Cas9, Rosa26-mT/mG.<br>- AZD4547 intervention: FVB/NRj WT mice, 8-week-old.<br>- PDXs: BALB/cAnNRj-Foxn1nu/nu or NOD.CB17-Prkdcscid/NCrHsd mice, 8-week-old.<br>The maximal permitted disease endpoints were not exceeded in any of the experiments. The mouse colony was housed in a certified animal facility with a 12-hour light/dark cycle in a temperature- and humidity-controlled room set to 21 °C and 55% relative humidity. Mice were kept in individually ventilated cages, and food and water were provided ad libitum.
Wild animals	No wild animals were used in the study.
Field-collected samples	No field collected samples were used in the study.
Ethics oversight	GEMMs and somatic mouse models: All animal experiments were approved by the Animal Ethics Committee of the Netherlands Cancer Institute and performed in accordance with institutional, national and European guidelines for Animal Care and Use.<br>PDXs: All the procedures related to animal handling, care, and the treatment in this intervention study were performed according to guidelines approved by the Institutional Animal Care and Use Committee (IACUC) of Crown Bioscience following the guidance of the Association for Assessment and Accreditation of Laboratory Animal Care (AAALAC).

Note that full information on the approval of the study protocol must also be provided in the manuscript.

Human research participants

Policy information about studies involving human research participants

Population characteristics	The FIGHT-202 trial was previously published. Abou-Alfa, G. K. et al. Pemigatinib for previously treated, locally advanced or metastatic cholangiocarcinoma: a multicentre, open-label, phase 2 study. Lancet Oncol. 21, 671–684 (2020). Population characteristics are described in detail in Abou-Alfa et al., 2020, and its appendix. Briefly, the trial was done at 146 academic or community-based sites in the USA, Europe, the Middle East, and Asia. Age, median (range), 59 (26 to 78). Sex, male, 42%; female, 58%. Region, North America, 61%; Western Europe, 24%; Rest of world, 15%. Race, White, 71%; Asian, 15%; Black or African American, 6%; American Indian or Alaska Native, 1%; Other or data missing, 8%. Metastatic disease, 86%; Previous cancer surgery, 33%; Previous radiotherapy, 23%; Previous systemic therapies, 100%.
Recruitment	Details on patient recruitment were previously published (Abou-Alfa et al., 2020). Briefly, patients were identified during routine clinical practice. Eligible patients were aged 18 years or older and had a histological or cytological diagnosis of locally advanced or metastatic cholangiocarcinoma with documented disease progression following at least one previous systemic cancer therapy. Before assessment for eligibility, patients were pre-screened centrally for FGF/FGFR status using massively parallel DNA-sequencing (FoundationOne). Patients who already had an FGF/FGFR status report based on local assessment or an existing FoundationOne report were also included. Retrospective central confirmation of locally documented FGF/FGFR status with FoundationOne was required for cohort assignment. Based on the centrally confirmed results, patients were assigned to one of three cohorts: patients with FGFR2 fusions/REs, patients with other FGF/FGFR alterations, or patients with no FGF/FGFR alterations. No self-selection or other biases were observed.

Ethics oversight	The study protocol (see Abou-Alfa et al., 2020, appendix p26) was approved by each institutional review board or independent ethics committee; the trial was performed in accordance with the Declaration of Helsinki. Patients gave written, informed consent for inclusion in the study.

Note that full information on the approval of the study protocol must also be provided in the manuscript.

Clinical data

Policy information about clinical studies

All manuscripts should comply with the ICMJE guidelines for publication of clinical research and a completed CONSORT checklist must be included with all submissions.

Clinical trial registration	FIGHT-202 (NCT02924376)
Study protocol	The trial was previously published. Abou-Alfa, G. K. et al. Pemigatinib for previously treated, locally advanced or metastatic cholangiocarcinoma: a multicentre, open-label, phase 2 study. Lancet Oncol. 21, 671–684 (2020).<br><br>Study protocol is in the appendix (p26) of Abou-Alfa et al., 2020.<br>INCB 54828-202
Data collection	Data collection is described in Abou-Alfa et al., 2020 and its appendix.
Outcomes	Primary and secondary outcomes are described in Abou-Alfa et al., 2020.<br>The primary endpoint was the proportion of patients with FGFR2 fusions or rearrangements who achieved an objective response (best overall response of confirmed complete response or confirmed partial response), assessed by independent central review. Secondary endpoints were the proportion of patients with an objective response in patients with other FGF/FGFR alterations, in all patients with FGF/FGFR alterations, and in patients with no FGF/FGFR alterations, and duration of response, the proportion of patients with disease control, progression-free survival, overall survival, safety in all cohorts, and population pharmacokinetics (data to be reported separately). Progression-free survival was defined as the time from first dose to progressive disease or death, overall survival was defined as the time from first dose to death from any cause, duration of response was defined as the time from complete response or partial response to progressive disease or death, and disease control was defined as complete response, partial response, or stable disease.

Flow Cytometry

Plots

Confirm that:

☒ The axis labels state the marker and fluorochrome used (e.g. CD4-FITC).

☒ The axis scales are clearly visible. Include numbers along axes only for bottom left plot of group (a 'group' is an analysis of identical markers).

☒ All plots are contour plots with outliers or pseudocolor plots.

☒ A numerical value for number of cells or percentage (with statistics) is provided.

Methodology

Sample preparation	Mammary glands of Rosa26-mT/mG female mice injected with Fgfr2-P2A-Cre lentiviruses were minced and digested with 4 mg/ml collagenase A (#11088793001, Roche) and 25 ug/ml DNase I (#DN25, Sigma Aldrich) in Dulbecco's Modified Eagle Medium/Nutrient Mixture F-12 (DMEM/F-12, #31331, Thermo Fisher Scientific) containing 100 IU/ml penicillin and streptomycin (Pen Strep, #15070, Thermo Fisher Scientific) for 1 hr at 37 °C. Digests were passed through a 70 um cell strainer prewetted with PBS containing 10% Foetal Bovine Serum (FBS, #S-FBS-EU-015, Serana) and 2 mM EDTA (FACS buffer). Single cells were stained with BV650-conjugated anti-EpCAM antibody (1:100, #740559, BD Biosciences) in FACS buffer, labelled with the LIVE/DEAD Fixable Violet Dead Cell Stain Kit (405 nm excitation, #L34964, Thermo Fisher Scientific), fixed with BD Phosflow Fix Buffer I (#557870, BD Biosciences), and permeabilised with BD Phosflow Perm Buffer III (#558050, BD Biosciences), each for 30 min at 4 °C. Cells were incubated with primary antibodies overnight and subsequently with secondary antibodies for 1 hr both in FACS buffer and at 4 °C.<br>Cultured NMuMG cells were collected with 2mM EDTA and passed through a 70 um cell strainer prewetted with FACS buffer. Single cells were labelled with the LIVE/DEAD Fixable Violet Dead Cell Stain Kit and fixed with BD Phosflow Fix Buffer I, and permeabilised with BD Phosflow Perm Buffer III, each for 30 min at 4 °C. Cells were incubated with primary antibodies overnight and subsequently with secondary antibodies for 1 hr both in FACS buffer and at 4 °C.<br><br>Antibodies used are the following:<br>Rat monoclonal anti-EpCAM, BV650-conjugated, BD Biosciences, #740559, , lot#1187955, clone#G8.8, 1:100<br>Rabbit monoclonal anti-FGFR2, Cell Signaling Technology, #11835, lot#4 and 5, clone#D4H9, 1:200<br>Goat polyclonal anti-GFP, Abcam, #ab6673, lot#GR3371856-3, 1:200<br>Donkey polyclonal anti-goat IgG (H+L), AF488-conjugated, Thermo Fisher Scientific, #A-11055, lot#2301114, 1:400<br>Donkey polyclonal anti-rabbit IgG (H+L), AF647-conjugated, Thermo Fisher Scientific, #A32795, lot#WA308388, 1:400
Instrument	BD LSRFortessa Cell Analyzer (BD Biosciences) equipped with 405 nm (450/50, 670/30 pass filters), 488 nm (530/30 pass filters), and 638 nm (670/30 pass filters) lasers.

Software	Data were analysed with BD FACSDiva Software (version 8.0.2, BD Biosciences) and FlowJo (version 10.7.1, BD Biosciences).
Cell population abundance	FACS sorting was not performed, thus post-sorting purity of fractions was not assessed.
Gating strategy	NMuMG cells were gated for (i) FSC-A / SSC-A to select bulk of cells and exclude debris events, (ii) SSC-A / SSC-H to select single cells, (iii) FSC-A / 405-Live/Dead to select live cells, (iv) FSC-A / FGFR2, AF647 to gate and subsequently display FGFR2 intensity as histogram and measure FGFR2 MFI. See Supplementary Figure 1a.<br>Mammary gland-derived cells were gated for (i) FSC-A / SSC-A to select bulk of cells and exclude debris events, (ii) SSC-A / SSC-H to select single cells, (iii) FSC-A / 405-Live/Dead to select live cells, (iv) FSC-A / BV650-EpCAM to select EpCAM+ cells, (v) FSC-A / EGFP, AF488 to select EGFP- and EGFP+ cells, (vi) FSC-A / FGFR2, AF647 for gating (not shown) to subsequently display FGFR2 intensity as histogram and measure FGFR2 MFI of EGFP- and EGFP+ cells. See Supplementary Figure 1b.

☒ Tick this box to confirm that a figure exemplifying the gating strategy is provided in the Supplementary Information.
